# Pol II–Expressed shRNA Knocks Down *Sod2* Gene Expression and Causes Phenotypes of the Gene Knockout in Mice

**DOI:** 10.1371/journal.pgen.0020010

**Published:** 2006-01-27

**Authors:** Xu-Gang Xia, Hongxia Zhou, Enrique Samper, Simon Melov, Zuoshang Xu

**Affiliations:** 1 Department of Biochemistry and Molecular Pharmacology, University of Massachusetts Medical School, Worcester, Massachusetts, United States of America; 2 Buck Institute for Age Research, Novato, California, United States of America; 3 Cell Biology, University of Massachusetts Medical School, Worcester, Massachusetts, United States of America; 4 Neuroscience Program, University of Massachusetts Medical School, Worcester, Massachusetts, United States of America; Harvard Medical School, United States of America

## Abstract

RNA interference (RNAi) has been used increasingly for reverse genetics in invertebrates and mammalian cells, and has the potential to become an alternative to gene knockout technology in mammals. Thus far, only RNA polymerase III (Pol III)–expressed short hairpin RNA (shRNA) has been used to make shRNA-expressing transgenic mice. However, widespread knockdown and induction of phenotypes of gene knockout in postnatal mice have not been demonstrated. Previous studies have shown that Pol II synthesizes micro RNAs (miRNAs)—the endogenous shRNAs that carry out gene silencing function. To achieve efficient gene knockdown in mammals and to generate phenotypes of gene knockout, we designed a construct in which a Pol II (ubiquitin C) promoter drove the expression of an shRNA with a structure that mimics human miRNA miR-30a. Two transgenic lines showed widespread and sustained shRNA expression, and efficient knockdown of the target gene *Sod2*. These mice were viable but with phenotypes of SOD2 deficiency. Bigenic heterozygous mice generated by crossing these two lines showed nearly undetectable target gene expression and phenotypes consistent with the target gene knockout, including slow growth, fatty liver, dilated cardiomyopathy, and premature death. This approach opens the door of RNAi to a wide array of well-established Pol II transgenic strategies and offers a technically simpler, cheaper, and quicker alternative to gene knockout by homologous recombination for reverse genetics in mice and other mammalian species.

## Synopsis

Reverse genetics studies gene functions by altering a gene and observing the consequences. A powerful method of reverse genetics in mammals is gene knockout by homologous recombination, which mutates a gene to prevent its functional expression. Using this method, investigators have revealed the functions of many genes. However, this method is relatively complex, time-consuming, and costly. In addition, this method is limited to studies in mice because it is not well established in other mammalian species. The authors of this study tested an alternative method using RNA interference (RNAi), which is a widely conserved mechanism in eukaryotes and can mediate gene-specific silencing. These investigators used RNA polymerase II (Pol II) to express a short hairpin RNA (shRNA) that triggers destruction of the mRNA-encoding Mn superoxide dismutase (SOD2) in transgenic mice. These mice exhibit phenotypes that were typical in *Sod2* knockout mice, including elevated levels of oxidative stress in various tissues, fat deposition in liver and muscles, dilated cardiomyopathy, and premature death. These results open the door of RNAi to a wide array of well-established Pol II transgenic strategies and offer a technically simpler, cheaper, and quicker alternative to gene knockout for reverse genetics in mice and other mammalian species.

## Introduction

Gene knockout by homologous recombination has been instrumental in investigating gene functions in mammals. It has been used to reveal gene functions in normal as well as in pathogenic pathways in vivo, and to generate models for many genetic disorders. However, the technical complexity, lengthy process, and high cost have limited its broad application. This is particularly problematic considering the fact that of the ~30,000 currently known mouse genes, only 10% have been knocked out, and even fewer are readily accessible by the research community [1]. Furthermore, the knockout technology, in most instances, generates animals with ~50% or 0% expression of the target gene, and it is difficult to create graded hypomorphic models, which may be necessary for modeling some diseases. In addition, the knockout technology is not well established in other mammalian species, consequently limiting the investigation of gene functions and the development of disease models in other mammalian species. These limitations may be overcome by RNA interference (RNAi) technology [1,2].

RNAi is a widely conserved mechanism in eukaryotes [3]. Triggered by double-stranded RNA (dsRNA) in cells, RNAi destroys the target RNA that shares sequence homology with the dsRNA [4]. The mechanism of RNAi is not fully understood. A simplified model based mainly on data from *Drosophila* has the following steps: First, Dicer, an enzyme of the RNase III family, initiates ATP-dependent fragmentation of long dsRNA into 21- to 25-nucleotide double-stranded fragments, called small interfering RNAs (siRNAs). Second, the siRNA duplexes bind the proteins Dicer and R2D2, which facilitate the formation of a siRNA/multiprotein complex called RNA-induced silencing complex (RISC) loading complex. Third, the siRNA duplex in the RISC loading complex unwinds to form an active RISC that contains a single-stranded RNA (called the guide strand). Fourth, the RISC recognizes the target RNA by Watson–Crick base pairing with the guide strand and cleaves the target RNA. Finally, the RISC releases its cleaved product and goes on to catalyze a new cycle of target recognition and cleavage [5].

In differentiated mammalian cells, long dsRNA activates RNA-dependent protein kinase PKR and type I interferon response, which leads to a nonspecific global translation depression and apoptosis [6]. However, this nonspecific reaction can be circumvented by introduction of synthetic siRNA [7,8], which can go into the RNAi pathway much like the siRNAs produced from long dsRNA can. Alternatively, RNAi may be triggered by a short hairpin RNA (shRNA) synthesized from gene constructs with RNA polymerase III (Pol III) or Pol II promoters [9]. The Pol III–synthesized shRNA is exported by exportin 5 from the nucleus to the cytoplasm [10,11], where it is processed by Dicer to generate siRNA, which goes into the RNAi pathway and mediates gene silencing. The Pol II–expressed shRNA mimics micro RNAs (miRNAs) and has one additional step of processing before nuclear export: it is synthesized as a long Pol II transcript [12,13] and is processed by the microprocessor complex that contains Drosha and Pasha to produce the shRNA [14,15].

The simplicity and specificity of RNAi has made RNAi a routine tool for investigation of gene functions in invertebrates and mammalian cells. Attempts have also been made to develop RNAi as an in vivo reverse genetics tool in mice. An early experiment directly injected long dsRNA into mouse embryonic stem cells. Successful knockdown and the phenotype of the gene deletion were observed in embryogenesis [16]. This approach can produce only transient inhibition of target gene expression. Another experiment used an oocyte-specific promoter to express a long hairpin (~500 bp) against c-Mos and demonstrated the expected phenotypes in oogenesis [17]. Both approaches cannot be applied widely because of the toxicity associated with long dsRNA in differentiated somatic cells.

With the advent of Pol III promoter-directed synthesis of shRNAs, several groups engineered transgenic mice using Pol III–shRNA constructs [18–23]. In all cases, knockdown of the marker transgene GFP in neonates was reported. In two cases, phenotypes resembling genetic knockouts were observed in developing mouse embryos [18] and hematopoietic stem cells [19]. Recently, Cre-lox–inducible Pol III promoters have been demonstrated to knock down target genes and induce phenotypes of gene deficiency in developing mouse embryos [24–26]. However, the success rate of gene knockdown and phenotypic expression is low using Pol III constructs [22,27]; despite the improvements using approaches employing targeted insertion in embryonic stem cells and tetraploid blastocyst complementation [28], widespread knockdown and phenotypes of gene deletion that develop in postnatal animals have not been demonstrated.

In addition to Pol III, Pol II can also direct shRNA synthesis [29,30] and mediate efficient silencing in cultured cells [13]. Compared with Pol III promoters, Pol II promoter-directed synthesis of shRNAs can be advantageous for transgenic RNAi. Although limited choices of Pol III promoters have been developed to express shRNA, a large repertoire of Pol II promoters, including temporally and spatially specific and inducible promoters, have been successfully used in transgenic mice. In addition, the current Pol III strategies rely on the availability of various Cre transgenic mouse lines for shRNA induction [24–26]. These Cre transgenic lines do not exist in other mammalian species. Therefore, the application of this Pol III strategy in other mammalian species is currently impractical. Recent evidence indicates that miRNAs, the endogenous form of shRNAs, are downstream of Pol II promoters [31] and are expressed by Pol II activity [32–34]. Therefore, strategies using Pol II–directed synthesis of shRNA mimic the natural miRNA synthesis and could be an efficient RNAi strategy in vivo.

To test this idea, we used a construct that is composed of a human ubiquitin C promoter and an shRNA with the human miRNA miR-30a structure [13] to generate transgenic mice. We targeted the shRNA against the mouse Mn superoxide dismutase *(Sod2)* gene, because *Sod2*-null mice generated by standard knockout technology have striking postnatal phenotypes that have been well characterized [35–37]. This Pol II strategy expressed siRNA widely in different tissues, knocked down the *Sod2* gene expression, and induced SOD2-hypomorphic phenotype. In addition, bigenic heterozygous mice generated by crossing the two independent transgenic lines showed phenotypes typical of the SOD2-null mouse, including slow growth, fatty liver, dilated cardiomyopathy, and premature death. Thus, this Pol II RNAi strategy can be an alternative to gene knockout technology and opens the door of RNAi to a wide array of Pol II transgenic strategies to investigate gene functions in mice and other mammalian species.

## Results/Discussion

**Figure 1:**
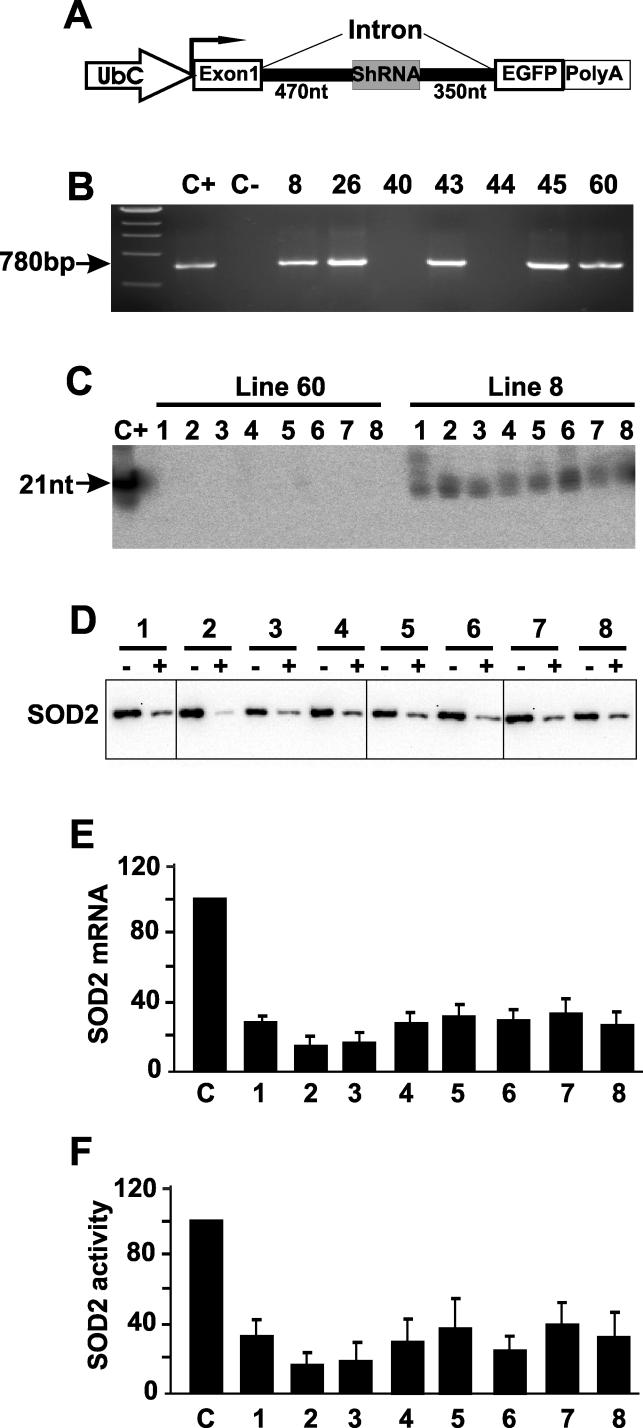
shRNA Expression and Knockdown of *Sod2* Gene Expression In Vivo (A) Schematic illustration of the transgene construct. The shRNA was designed to mimic human miR-30a structure (for details, see [13]). (B) PCR analysis of tail DNA identified transgenic founders. C+ indicates positive control; C−, negative control. Numbers indicate examples of various transgenic lines. (C) Northern blots detected shRNA expression in transgene-positive line-8 mice, but not in line-60 mice. Total RNA (30 μg) was loaded in each lane. The tissues are lung (1), heart (2), skeletal muscle (3), kidney (4), liver (5), brain (6), stomach (7), and spleen (8). C+ is the siRNA-positive control. (D) Western blots of SOD2 protein compare the SOD2 levels in the above tissues between line-8 mice and wild-type mice. Due to different levels of SOD2 in different tissues, different amounts of total protein from different tissues had to be loaded in order to maintain the assay in linear range. The amounts of proteins are the following in micrograms: (1) 30; (2–4) 10; (5–6) 15; (7) 20; and (8) 40. + indicates transgene positive; −, transgene negative. (E) SOD2 mRNA levels in the above tissues from transgenic line-8 mice measured by real-time PCR (*n* = 4; all “n” indicates mouse numbers). The levels were normalized to the level of SOD2 mRNA in tissues from the wild-type littermates, which were set as 100% (column C). (F) Levels of SOD2 activity in tissue lysates of transgenic line-8 mice compared with the wild-type littermates (*n* = 4).

The construct UbC-SOD2hp-EGFP (Figure 1; also see [13]) consists of the human ubiquitin C promoter, an shRNA-coding hairpin placed in the first intron, followed by the enhanced green fluorescent protein (EGFP) coding sequence and a poly-adenylation signal. The shRNA-coding hairpin mimics human microRNA miR-30a structure and target mouse *Sod2* mRNA. Using this construct, we generated transgenic mice by pronuclear injection of fertilized eggs. Screening of 62 founders yielded five positive lines (Figure 1A). Northern blots showed that two of the five lines (lines 8 and 26) expressed siRNA broadly (Figures 1B, 1C, and S1A) and this expression was stable over multiple generations and during aging (the same level of expression was observed in animals from 40 to 200 d old; unpublished data). By real-time PCR line 8 carried a single copy of the transgene while line 26 carried three copies (Figure S2). The transgene copy number did not predict the level of siRNA levels since line 8 had a higher level of siRNA than line 26 (see below).

The pattern of expression in transgenic mice differed from the pattern in cultured cells in two regards: none of these transgenic lines expressed detectable EGFP and shRNA (only siRNA was detected). This contrasts with what we observed in cultured cells, in which both were detectable [13]. Nevertheless, the siRNA knocked down the abundance of SOD2 as indicated by the decreased levels of the protein (Figures 1D and S1B), mRNA (Figures 1E and S1C), and enzyme activity (Figure 1F). The rapid processing of pre-miRNA in vivo probably caused the lack of shRNA detection. This explanation is consistent with the lack of pre-miRNA detection for other endogenous miRNAs in mammalian cells [34]. The lack of EGFP was puzzling. One possibility was that the transgene was altered. This was ruled out by sequencing the transgene extracted by PCR, which revealed no alteration in the transgene structure. Another possibility was that the pri-miRNA processing in vivo was highly efficient, so that the processing of pri-miRNA occurred before the splicing, and, consequently, the mRNA could not be properly spliced and exported to the cytoplasm for EGFP expression. The processing of pri-miRNA involves Drosha [14]. Therefore, if our hypothesis was correct, inhibition of Drosha expression should lead to EGFP expression. To test this, we transduced fibroblasts isolated from the skeletal muscle of the transgenic mice using a recombinant adenovirus that expresses an shRNA against Drosha. By RT-PCR the Drosha mRNA levels were substantially reduced (Figure S3A), indicating the effectiveness of the shRNA. While the nontransduced cells showed no detectable EGFP fluorescence (Figure S3B and S3C), the transduced cells expressed EGFP (Figure S3D and S3E). This result supports our hypothesis.

**Figure 2:**
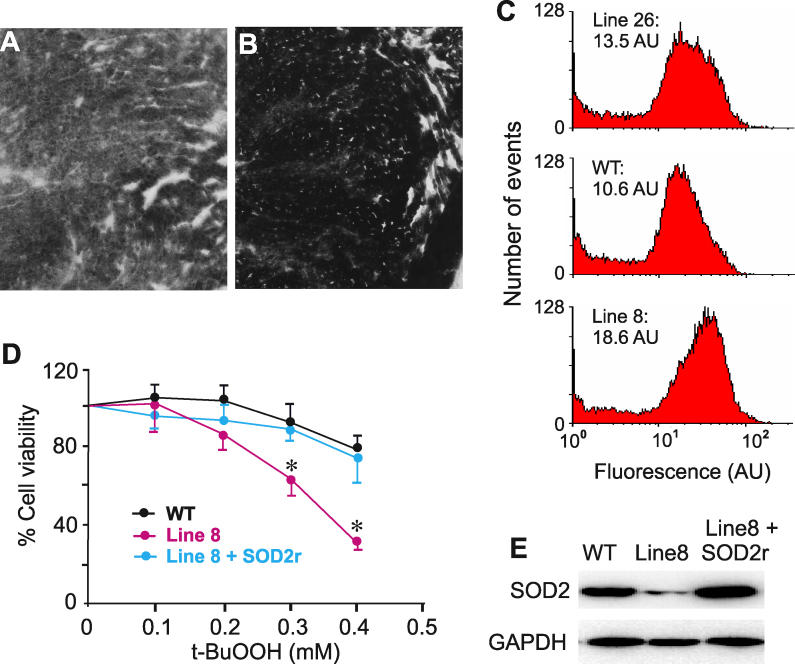
Consequence of the SOD2 Knockdown (A) Histochemical staining reveals that SDH activity in the heart of transgenic line-8 mice was reduced compared with the wild-type littermates (B). (C) ROS levels are increased in fibroblasts from the skeletal muscles of transgenic line-26 (top panel) and line-8 (bottom panel) mice, compared with those from the wild-type mice (middle panel). AU, arbitrary units. (D) Fibroblasts from the transgenic line-8 mice have elevated sensitivity to oxidative stress compared with those from the wild-type mice, and this sensitivity can be corrected by expressing an RNAi-resistant SOD2 (line 8 + SOD2r). The data are means observed in cells isolated from four individual mice. Error bars are SEM. The asterisks indicate significant difference as compared to either WT or rescued cells (*p* < 0.05). (E) Western blot detects SOD2 protein levels in fibroblasts isolated from the skeletal muscle of the wild-type and transgenic line-8 mice. The third lane is from the line-8 cells transduced with RAd expressing the siRNA-resistant SOD2.

To confirm the knockdown of *Sod2* gene expression, we examined the in vivo consequence of SOD2 deficiency. First, we compared the activity of the mitochondrial enzyme succinate dehydrogenase (SDH) between the transgenic and wild-type animals, because SOD2 knockout is known to cause a decrease in SDH activity [37,38]. We stained tissue sections from the heart using histochemical staining, and observed a decrease in the staining intensity in line 8 (Figure 2A and 2B). Second, we isolated fibroblasts from skeletal muscle and measured the levels of superoxide in these cells. We observed that the superoxide levels were increased in the two transgenic lines (Figure 2C). This increase was higher in line 8 than in line 26, and, therefore, was correlated with the degree of SOD2 knockdown (see below). Third, we tested the sensitivity of the fibroblasts to oxidative stress induced by t-butylhydroperoxide (t-BuOOH) treatment. Fibroblasts from transgenic line 8 showed a higher sensitivity than the wild-type cells (Figure 2D), consistent with the lowered levels of SOD2 protein in these cells (Figure 2E). To ensure that the enhanced sensitivity was caused by the knockdown of SOD2 expression, we transduced the fibroblasts with a recombinant adenovirus that expressed a *Sod2* gene whose mRNA was resistant to the siRNA expressed by the transgene. Expression of this SOD2 molecule (Figure 2E) rescued the cells from their hypersensitivity to oxidative stress (Figure 2D).

Based on these data, we conclude that ubiquitin C promoter–directed shRNA synthesis effectively silenced the target molecule in transgenic mice. Despite this significant knockdown (by 60%–90%) in all the tissues examined and the evidence of functional SOD2 deficiency, the two transgenic lines were viable to 400 d (observed to date). SOD2-null phenotypes, including small body size, dilated cardiomyopathy, lipid deposition in liver and heart, and premature death, were not observed. To determine whether knockout phenotypes could be generated, we crossed the two lines that expressed the siRNA to generate bigenic heterozygous transgenic mice. We took this approach because it was advantageous compared with generating homozygous animals of each lines, the phenotype of which could be complicated by the potential gene disruption at the transgene insertion site.

**Figure 3:**
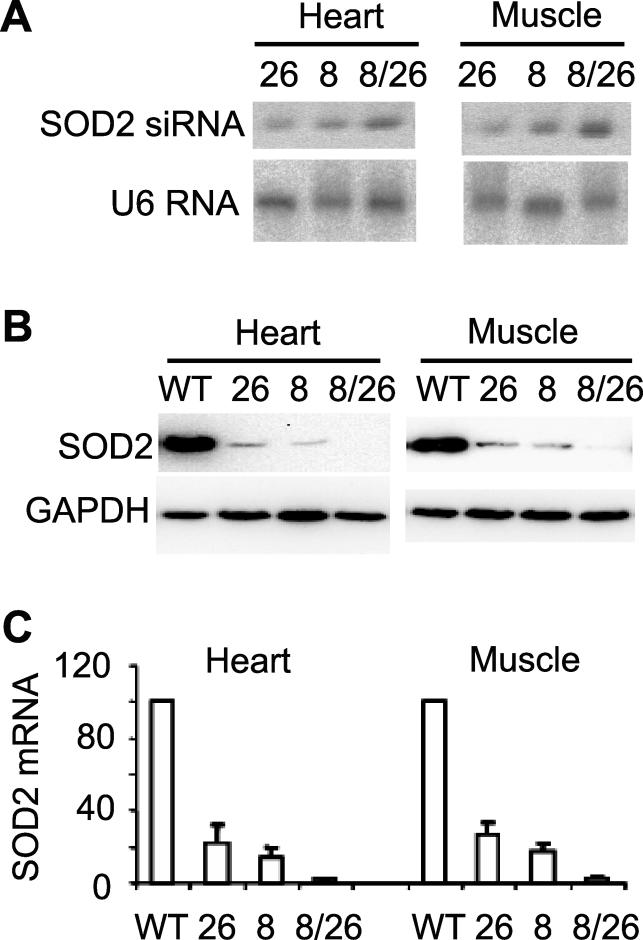
The Level of SOD2 Expression Was Knocked Down Further in Bigenic Transgenic Mice Generated by Crossing the Two Lines (Lines 8 and 26) (A) Northern blots indicate that siRNA levels are further increased in the 8/26 bigenic mice. (B) Western blots demonstrate that SOD2 protein levels are further knocked down in the 8/26 bigenic mice. (C) Real-time PCR shows that SOD2 mRNA levels are further lowered in the 8/26 bigenic mice.

**Figure 4:**
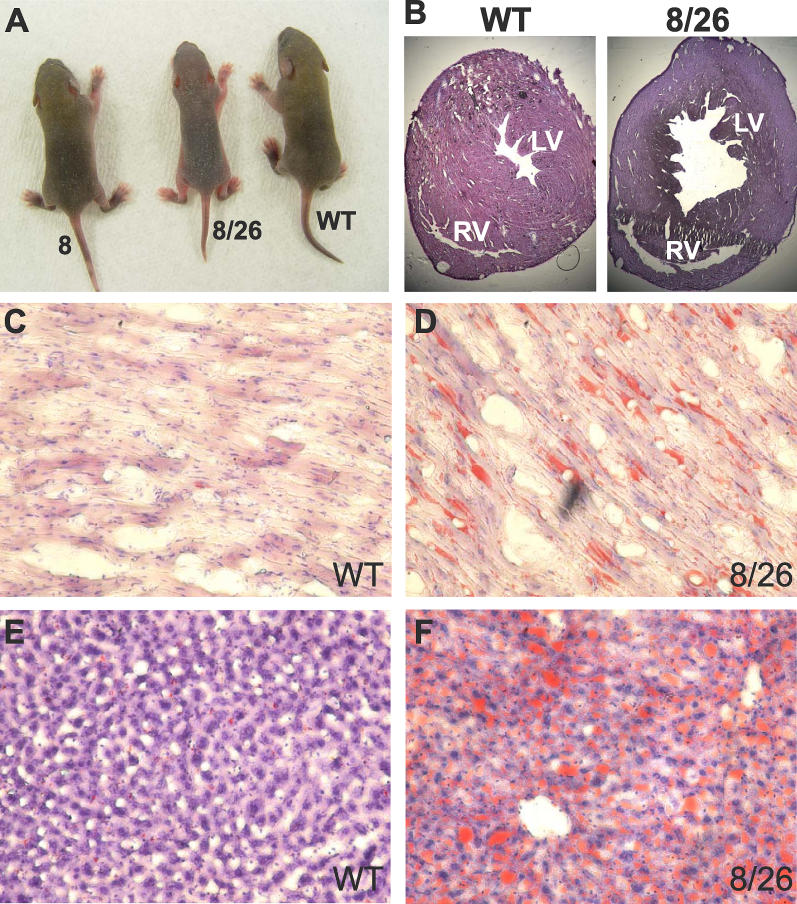
The 8/26 Bigenic Mice Display the Phenotype of a SOD2-Null Animal (A) Retarded growth (7-d-old animals). (B) Dilated cardiomyopathy (H&E-stained coronal sections of heart). LV, left ventricle; RV, right ventricle. (C–F) Lipid deposition in heart (C and D) and liver (E and F) stained with Oil Red O.

The line 8/26 bigenic mice expressed a higher level of siRNA than either of the singly transgenic lines (Figure 3A), leading to knockdown of SOD2 protein and mRNA to nearly undetectable levels (Figure 3B and 3C). These mice exhibited phenotypes similar to the previously reported SOD2 knockout mice [35], including smaller body size than the wild-type littermates (Figure 4A) and death within 20 d after birth (in 34 bigenic animals that we obtained so far). In addition, they developed dilated cardiomyopathy (Figure 4B) and had increased lipid deposition in the heart (Figure 4C and 4D) and the liver (Figure 4E and 4F).

**Figure 5:**
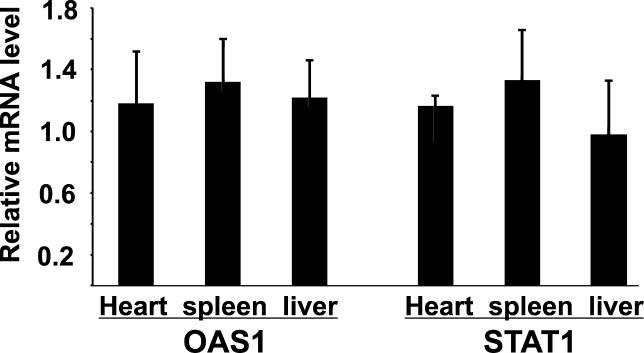
In Vivo Expression of SOD2 shRNA with Authentic miR30a Structure Did Not Up-Regulate the Expression of *OSA1* and *STAT1* Levels of the mRNAs were determined by real-time PCR. The levels of *OAS1* and *STAT1* mRNAs in the shRNA transgenic tissues from line-8 mice were normalized to *GAPDH* mRNA and expressed relative to that of wild-type littermate tissues. Data represent means from four mice + SEM.

Several studies have shown that some shRNA or siRNA could trigger interferon response [39–41]. This raises the possibility that interferon response might be responsible for the phenotype observed in these mice. To test this, we examined the levels of two genes known to be dramatically induced by the dsRNA-triggered interferon response, 2′,5′-oligoadenylate synthetase 1 *(OSA1),* and signal transducer and activator of transcription 1 *(STAT1)* [40,41]. Real-time PCR analysis of the mRNA of *OSA1* and *STAT1* failed to detect changes in the expression levels of these two genes in the heart, spleen, and liver in the transgenic line 8 (Figure 5), indicating no interferon response in these transgenic mice.

Taken together, the phenotypes are likely caused by the specific effect of SOD2 knockdown because (1) the siRNA was expressed widely; (2) the consequences of SOD2 deficiency were observed in the transgenic mice; (3) in cells isolated from these mice the hypersensitivity to oxidative stress was corrected by the *Sod2* gene that was resistant to the siRNA; (4) the phenotype typical of the SOD2-null mouse was observed when siRNA levels were increased in the bigenic 8/26 mice; and (5) levels of OSA1 and STAT1*,* two molecules involved in the dsRNA-induced interferon response, were unchanged. These results demonstrate that Pol II–mediated expression of shRNA in transgenic mice can be used to investigate gene functions in mammals.

Thus, the Pol II–directed synthesis of shRNA can be an alternative to gene knockout technology for reverse genetics in mammals. Although gene knockout remains a useful approach for the complete gene deletion or gene modification, our RNAi approach can achieve near knockout conditions and is economical in cost and time. In addition, the construct design is simple and in principle not different from the standard transgene design for gene overexpression. The placement of the shRNA-encoding hairpin is flexible: it can be placed in introns or in the 3′ untranslated regions [13,42], and the shRNA-encoding transcript is not required to encode a protein [13,29,30]. Therefore, a wide variety of transgene promoters that have already been successfully used to overexpress genes in transgenic mice can be readily adapted for suppressing the genes. When spatially and temporally specific promoters are used, controlled suppression of the target gene can be achieved. Furthermore, this approach provides a more flexible system to model hypomorphic allele function. While the standard knockout technology reduces the target gene expression to 50% or 0% in most instances, transgenic RNAi can reduce the target gene to more variable degrees in different lines of transgenic mice or by induction using small molecules in transgenic mice made with inducible Pol II promoters [43,44]. The simplicity of this approach can accelerate the generation of models for diseases that are caused by various degrees of genetic hypomorphism, such as autosomal dominant polycystic kidney disease [45], various cancers [46,47], and other diseases. Finally, gene knockout by homologous recombination has not been established in other mammalian species. Current Pol III strategies rely on the crosses with numerous Cre-expression transgenic mouse lines [24–26], which are not available in other mammalian species. Our knockdown strategy can overcome these limitations and be used to carry out reverse genetics and to generate disease models in other mammalian species.

## Materials and Methods

### Generation of SOD2 knockdown transgenic mice.

The transgene construct that contains the hairpin targeting the *Sod2* gene under the control of human ubiquitin C promoter (UbC-SOD2hp-EGFP) has been described previously [13]. The transgenic mice were made by pronuclear injection of the linearized construct into the fertilized eggs, which were generated from crossings of C57BL/6 and SJL. Positive founders and offspring carrying the transgene were identified by PCR of the tail DNA using the primers 5′-CGCCCTCGTCGTGTCGTGA-3′ (forward) and 5′-CGGCGCGGGTCTTGTAGTTGC-3′ (reverse). The transgenic lines were maintained by crossing founders to C57BL/6. To generate bigenic heterozygous transgenic mice, two shRNA-expressing lines were crossed and the doubly transgenic mice were identified by quantitative real-time PCR of tail DNA.

### Northern blot.

Mice were decapitated under anesthesia, and various tissues were quickly dissected, snap-frozen in liquid nitrogen, and stored at −80 °C. The total RNA was extracted from frozen mouse tissues using Trizol (Sigma, St Louis, Missouri, United States). Thirty micrograms of total RNA was fractionated on 15% polyacrylamide gels and transferred onto Hybond TM-N+ membrane (Amersham Biosciences, Little Chalfont, United Kingdom). After UV cross-linking, the membrane was probed with ^32^P-labeled synthetic RNA oligonucleotide complementary to the antisense strand of the mouse *Sod2* shRNA as described previously [13]. For some blots, the membranes were reprobed with ^32^P-labeled synthetic DNA oligonucleotide (5′-ACGAATTTGCGTGTCATCCTTGCG-3′) complementary to mouse U6 RNA.

### Western blot.

The frozen mouse tissues were homogenized in ice-cold lysis buffer containing 0.4% NP-40, 0.2 mM Na_3_VO_4_, 20 mM HEPES (pH 7.9), and a cocktail of protease inhibitors (Complete-Mini; Sigma). The protein content in the cleared lysate was determined using the BCA assay. Equal amount of total proteins from transgenic and wild-type control animals was resolved by 15% SDS-PAGE and blotted onto GeneScreen Plus membrane (PerkinElmer, Wellesley, Massachusetts, United States). Proteins were detected using specific primary antibodies and the SuperSignal kit (Pierce Biotechnology, Rockford, Illinois, United States) and photographed using the Kodak Digital Image Station 440CF. The primary antibodies were: rabbit anti-Mn superoxide dismutase (SOD2; 1:1,000, Stressgen Biotechnologies, San Diego, California, United States) and mouse anti–glyceraldehye-3-phosphate dehydrogenase (GAPDH; 1:10,000; Abcam, Cambridge, United Kingdom). After detection of SOD2, the membrane was stripped for 30 min at 55 °C in a buffer containing 100 mM β-mercaptoethanol, 2% SDS (w/v), and 62.5 mM Tris-HCl (pH 6.7), and used again for detection of GAPDH immunoreactivity, which served as loading control.

### Real-time quantitative RT-PCR.

Total RNA isolated using the TRI reagent was further purified with the RNeasy kit (Qiagen, Valencia, California, United States) and subjected to digestion on column with RNase-Free DNase (Qiagen). One microgram of purified total RNA from each sample was reversely transcribed to cDNA with oligo-dT primer using the RT kit (Invitrogen, Carlsbad, California, United States). The cDNA was used for quantitative PCR with SYBR green kit (Qiagen) according to manufacturer's instruction. The primer concentration was 500 nM. Cycling conditions were 15 min at 95 °C (to activate the hot-start Taq polymerase supplied with the SYBR Green detection kit), followed by 40 cycles of 15 s at 94 °C, 30 s at 60 °C, and 20 s at 72 °C. During amplification the fluorescence signal, which is proportional to the amount of dsDNA produced, was monitored. A complete amplification profile for each of the 96 wells of a PCR plate was obtained, which was used for the analysis. At the end of the PCR run, melting curves of the amplified products were obtained, which were used to determine the specificity of the amplification reaction. In pilot experiments, aliquots of the amplified products were separated on 3% agarose gels to ensure amplification of specific products of the predicted length. The amplification curves were used to calculate the threshold cycle number at which the amplification curve reaches the beginning of the linear phase of amplification. The threshold cycle number for *Sod2* gene was normalized to those of the housekeeping genes *GAPDH* and ribosomal RNA L17. The knockdown of the *Sod2* expression was determined by calculating the fold change of *Sod2* in transgenic tissues relative to the wild-type tissues.

### SOD2 activity.

SOD2 activity was determined by inhibition of xanthine/xanthine oxidase–induced cytochrome C reduction [48]. Frozen tissues were homogenized in 10 volumes of ice-cold buffer (10 mM KH_2_PO_4_ [pH 7.4], 20 mM EDTA, 30 mM KCl). The supernatant was collected after centrifugation of homogenates in a desktop microcentrifuge for 15 min at 4 °C and measured for protein concentration using BAC assay (Pierce). The activity of SOD1 was inactivated by treating the lysates with 5 mM KCN before SOD2 activity assay. To a cuvette, 967 μl of solution A (50 μM xanthine, 20 μM acetylated cytochrome C, 25 μM KH_2_PO_4_, 25 μM Na_2_HPO_4_, and 0.1 mM EDTA) was added, followed by 16.7 μl of the treated lysates and 16.7 μl of solution B (0.2 U/ml xanthine oxidase in 0.1 mM EDTA). The absorbance at 550 nm was read at 1-min intervals for 10 min. SOD2 activity in knockdown lysates was determined by comparing with an SOD2 standard curve (SOD2 enzyme; Sigma) and expressed relative to that in the wild-type lysates.

### SDH histochemistry.

Immediately following death, tissues were harvested and frozen on dry ice and sectioned at 20 μm thickness at −25 °C. SDH staining was performed and evaluated as previously described on frozen sections [38].

### Evaluation of ROS levels and tolerance to oxidative stress in fibroblasts.

Fibroblasts were isolated from skeletal muscle in 15-d-old mice using the method modified from Crisona et al. [49]. Skeletal muscles were removed from hindlimbs and minced in Hank's balanced salt solution. The muscle slurry was digested with collagenase type I (0.5% in Hank's balanced salt solution; Invitrogen) at 37 °C for 1 h. The cells were pelleted by centrifugation and subjected to further digestion with trypsin at 37 °C for 1 h. After another round of centrifugation and washing, the cells were plated to 75-cm^2^ flasks and grown in DMEM supplemented with 10% FBS, 100 mg/ml streptomycin, and 100 U/ml penicillin. The cultured cells were split every 3 d and used before four passages. For determination of ROS (superoxide), 500,000 primary (passage 2) wild-type and *Sod2* shRNA transgenic skeletal muscle fibroblasts were incubated in DMEM media containing 10% fetal calf serum, antibiotics, and 2 μM dihydroethidine (Molecular Probes, Eugene, Oregon, United States) for 30 min at 37 °C in the dark. After incubation, the cells were centrifuged, resuspended in freshly prepared cold FACS staining buffer (PBS, 1% BSA), and the FL-2 fluorescence of 20,000 cells per sample was analyzed using a Becton Dickinson LSR cytometer (Palo Alto, California, United States). In the same experiments, passage-1 wild-type and *Sod2* ^−/−^ mouse embryonic fibroblasts were included as controls. To prevent bias, the ROS determination was performed in a blind fashion. For viability assay, the cells were plated to 96-well plates at 1 × 10^4^ cells per well and allowed to attach overnight at 37 °C. On the following day, the cells were treated with t-BuOOH at different concentrations for 2 h. After three washes with PBS, the cells were grown in DMEM containing 10% FBS and MTS reagent (Promega, Madison, Wisconsin, United States) for 2 h. To measure the viability, the absorbance of cultures was read at 495 nm with a plate reader and normalized to the average absorbance of the cells untreated with tBuOOH. For the rescue experiments, the transgenic cells (two passages) were infected in 25-cm^2^ flasks with adenoviral vectors expressing RNAi-resistant *Sod2* gene (see below). After 3 d, the transduced cells were split into 96-well plates, grown for 24 h, and treated with t-BuOOH; the viability was measured as described above. Statistical analysis was performed using ANOVA followed by Tukey post-hoc test to compare group means.

### Adenoviral vector expressing RNAi-resistant *Sod2.*

ViraPower adenoviral kit (Invitrogen) was used. An RNAi-resistant *Sod2* gene was generated by introducing four silent mutations within shRNA target region. The target sequence of SOD2 mRNA was 5′-AAGGGAGATGTTACAACT-3′, and the target sequence of RNAi-resistant SOD2 mRNA was 5′-AAG GG**T** GA**C** GT**A** AC**T** ACT-3′ (bold letters indicate the silent mutations). The CMV-SOD2 expression cassette was introduced into ViraPower adenoviral vector (RAd; Invitrogen) using the Gateway method. The RAd-SOD2 vector was transiently transfected into a RAd producer cell line 293A that stably expresses E1 proteins required for production of adenovirus. The RAd-SOD2 was harvested from transiently transfected 293A cells and amplified by infecting fresh 293A cells. The virus was purified by CsCl gradient centrifugation, dialyzed against a buffer containing 10 mM Tris (pH 7.5), 1 mM MgCl_2_, and 10% glycerol, and stored in aliquots at −80 °C. The viral titer (plaque-forming unit) was determined using 293A cells according to the manufacturer's instructions. To transduce fibroblast cells, the virus was used at a multiplicity of infection (MOI; 50) and incubated with the culture for 3 h before the media were changed. The transduced cells were used for viability assay at 48 h after transduction.

### Oil Red O staining.

Oil Red O staining was performed on frozen sections. The fresh tissues collected as described above were frozen in powdered dry ice. The frozen sections (12 μm) were cut using a Cryostat and stained with Oil Red O solution on slides as follows: the sections were fixed in 10% formalin for 5 min, washed with several changes of phosphate-buffered saline, stained in prewarmed Oil Red O solution (60 °C; Sigma) for 8 min, washed again several times with distilled water, stained in Gill's hematoxylin solution for 30 s, and washed several times in distilled water. After mounted with coverslips, the stained sections were observed under microscope and photographed.

## Supporting Information

Figure S1 shRNA Expression and Knockdown of *Sod2* Gene Expression in Line 26 (A) Northern blots detect shRNA expression in transgene-positive line 26. Total RNA (30 μg) was loaded in each lane. The tissues are lung (1), heart (2), skeletal muscle (3), kidney (4), liver (5), brain (6), stomach (7), and spleen (8).
(B) Western blots compare the SOD2 protein levels in the above tissues between line-26 mice and wild-type mice. + indicates transgene positive, and − indicates transgene negative. The amounts of proteins are loaded in the same order as described in Figure 2.
(C) SOD2 mRNA levels in the above tissues from transgenic line 26 measured by real-time PCR (*n* = 4). The levels were normalized to the level of SOD2 mRNA in tissues from the wild-type littermates, which were set as 100% (column C).
(46 KB PDF)

Figure S2 Determining the Copy Number of the Transgene by Real-Time PCR (A) Testing the specificity of the primers used for real-time PCR. HEK293 cells were used as reference for gene copy number of ubiquitin C. NT, nontransgenic.
(B) Estimation of UbC-SOD2hp-EGFP gene copy numbers. A 118-bp segment in human ubiquitin C promoter was amplified using a pair of specific primers. Also amplified was human and mouse *SOD1* gene using a pair of primers that are complementary to both genes. Both *Ubiquitin C* and *SOD1* are single-copy genes. The threshold cycle number value of *Ubiquitin C* was normalized against the *SOD1* detected in the same sample. The normalized value from HEK293 genomic DNA represents two copies of the *ubiquitin C* gene. By normalizing this value from mouse genomic DNA samples against the value from HEK293 cells, the estimates of copy numbers of the *ubiquitin C* transgene were obtained. The PCR primers used for human and mouse *Sod1* gene were 5′-GACCTGGGCAATGTGACTGCTG-3′ (forward) and 5′-CACCAGTGTACGGCCAATGATG-3′ (reverse); and for *ubiquitin C* promoter were 5′-CGCTGCTCATAAGACTCGGC-3′ (forward) and 5′-TTTCCTCGCCTGTTCCGCTC-3′ (reverse). Bars are averages from four to six animals.
(33 KB PDF)

Figure S3 Silencing Drosha Unblocked the Expression of *EGFP* Transgene in Muscle Fibroblasts (A) Drosha was knocked down by RNAi in the fibroblasts from wild-type and line-8 transgenic mice. The cells were transduced with adenoviral vectors expressing an shRNA against mouse Drosha (shRNA stem sequence: 5′-GGATGAAGATTTAGAGAGTTC-3′). Four days after transduction, the total RNA was extracted from the cells and used for RT-PCR to detect Drosha. The ribosomal RNA L17 was magnified in parallel as input control. The PCR was run for 28 cycles using the following primers for Drosha: 5′-GAGCCTAGAGGAAGCCAAACA-3′ (forward) and 5′-GCCGGACGTGAGTGAAGAT-3′ (reverse); for L17: 5′-CGGTATAATGGTGGAGTTG-3′ (forward) and 5′-ACCCTTAAGTTCAGCGTTACT-3′ (reverse).
(B) No EGFP fluorescence could be detected in fibroblasts isolated from line-8 transgenic mice.
(C) The same field as in (B) was stained with DAPI.
(D) Three days after the fibroblasts were transduced with an adenoviral vector that expressed an shRNA against mouse Drosha, EGFP fluorescence was detected.
(E) The same field as in (D) was stained with DAPI.
(48 KB PDF)

## Abbreviations

dsRNA: double-stranded RNA
EGFP: enhanced green fluorescent protein
miRNA: micro RNA
Pol: RNA polymerase
RISC: RNA-induced silencing complex
RNAi: RNA interference
SDH: succinate dehydrogenase
shRNA: short hairpin RNA
siRNA: small interfering RNA
