# Supplementary material for: Pol II–Expressed shRNA Knocks Down Sod2 Gene Expression and Causes Phenotypes of the Gene Knockout in Mice
Source: PLoS Genet. 2006 Jan 27;2(1):e10. doi: 10.1371/journal.pgen.0020010 (PMC1358942; doi:10.1371/journal.pgen.0020010)
Supplement: Figure S1 — (A) Northern blots detect shRNA expression in transgene-positive line 26. Total RNA (30 μg) was loaded in each lane. The tissues are lung (1), heart (2), skeletal muscle (3), kidney (4), liver (5), brain (6), stomach (7), and spleen (8). (B) Western blots compare the SOD2 protein levels in the above tissues between line-26 mice and wild-type mice. + indicates transgene positive, and − indicates transgene negative. The amounts of proteins are loaded in the same order as described in Figure 2. (C) SOD2 mRNA levels in the above tissues from transgenic line 26 measured by real-time PCR (n = 4). The levels were normalized to the level of SOD2 mRNA in tissues from the wild-type littermates, which were set as 100% (column C). (46 KB PDF) [file pgen.0020010.sg001.pdf]

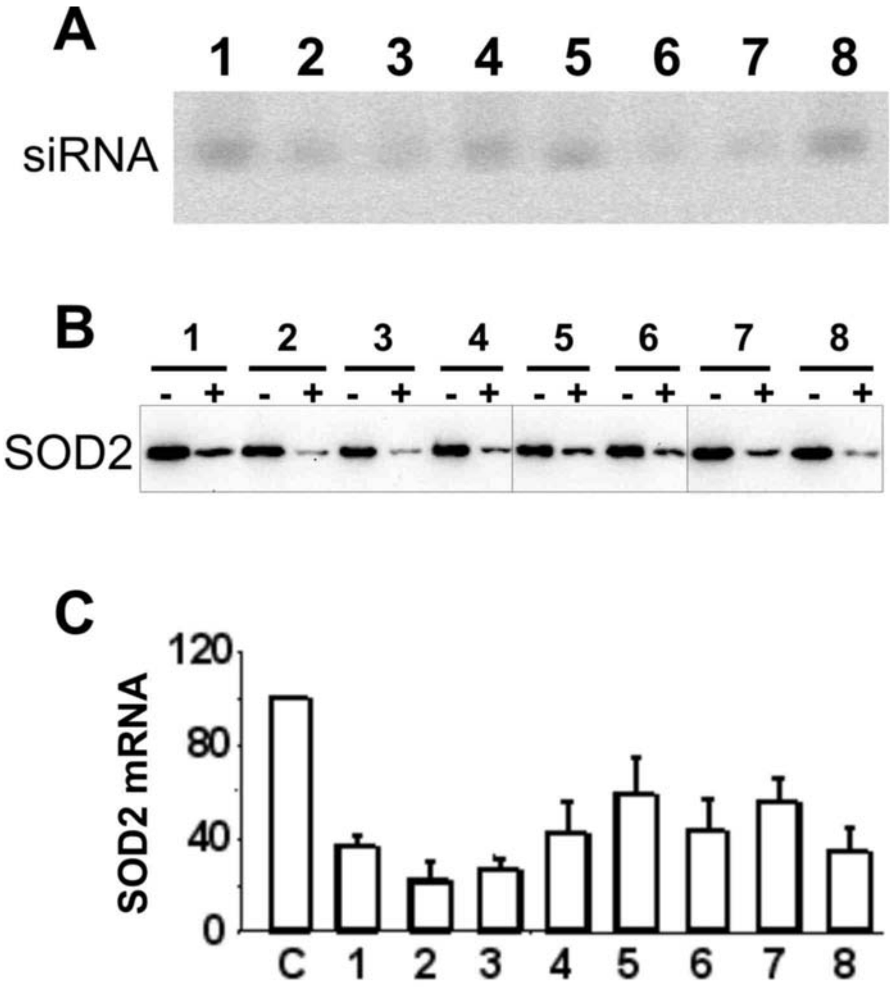

Supplemental figure 1. shRNA expression and knockdown of the target *Sod2* gene in line 26. (A) Northern blots detect shRNA expression in transgene-positive line 26. Thirty mg of total RNA were loaded in each lane. The tissues are lung (1), heart (2), skeletal muscle (3), kidney (4), liver (5), brain (6), stomach (7), and spleen (8). (B) Western blots of SOD2 protein compare the SOD2 levels in the above tissues between line 26 mice and wild type mice. "+" indicates transgene-positive and "-" indicates transgene-negative. The amounts of proteins from the different tissues are the same as described in figure 2. (C) SOD2 mRNA levels in the above tissues from transgenic line 26 measured by Realtime PCR (n = 4). The levels were normalized to the level of SOD2 mRNA in tissues from the wild type littermates, which were set as 100% (column C).
