# Supplementary material for: Pol II–Expressed shRNA Knocks Down Sod2 Gene Expression and Causes Phenotypes of the Gene Knockout in Mice
Source: PLoS Genet. 2006 Jan 27;2(1):e10. doi: 10.1371/journal.pgen.0020010 (PMC1358942; doi:10.1371/journal.pgen.0020010)
Supplement: Figure S2 — (A) Testing the specificity of the primers used for real-time PCR. HEK293 cells were used as reference for gene copy number of ubiquitin C. NT, nontransgenic. (B) Estimation of UbC-SOD2hp-EGFP gene copy numbers. A 118-bp segment in human ubiquitin C promoter was amplified using a pair of specific primers. Also amplified was human and mouse SOD1 gene using a pair of primers that are complementary to both genes. Both Ubiquitin C and SOD1 are single-copy genes. The threshold cycle number value of Ubiquitin C was normalized against the SOD1 detected in the same sample. The normalized value from HEK293 genomic DNA represents two copies of the ubiquitin C gene. By normalizing this value from mouse genomic DNA samples against the value from HEK293 cells, the estimates of copy numbers of the ubiquitin C transgene were obtained. The PCR primers used for human and mouse Sod1 gene were 5′-GACCTGGGCAATGTGACTGCTG-3′ (forward) and 5′-CACCAGTGTACGGCCAATGATG-3′ (reverse); and for ubiquitin C promoter were 5′-CGCTGCTCATAAGACTCGGC-3′ (forward) and 5′-TTTCCTCGCCTGTTCCGCTC-3′ (reverse). Bars are averages from four to six animals. (33 KB PDF) [file pgen.0020010.sg002.pdf]

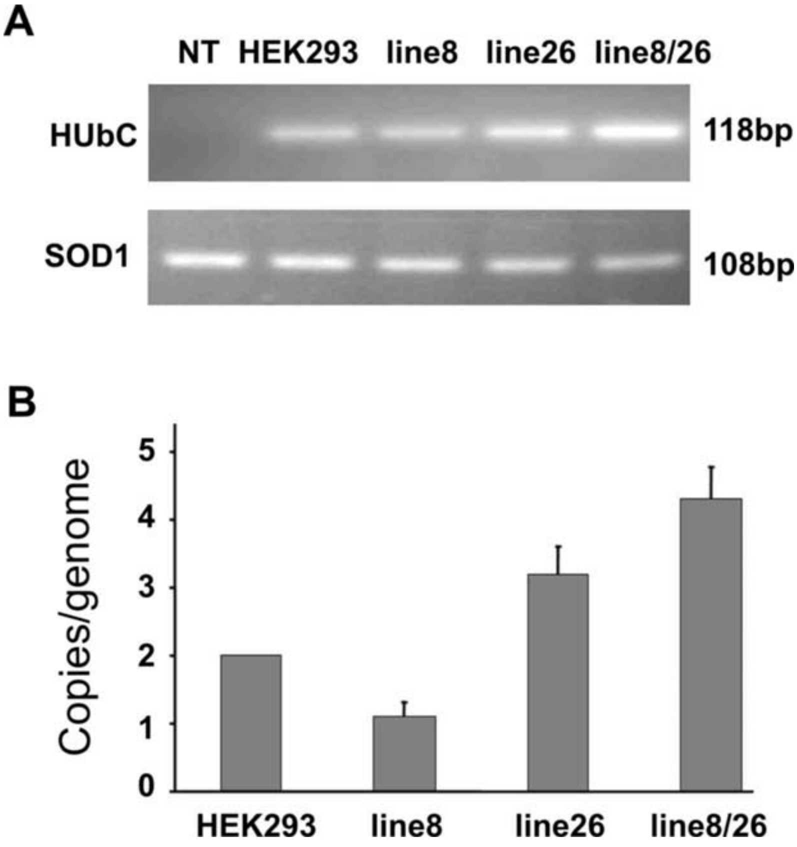

Supplemental figure 2. Determining the copy number of the transgene by Real-time PCR. (A) Testing the specificity of the primers used for real-time PCR. HEK293 cells were used as reference for gene copy number of ubiquitin C. NT means non-transgenic. (B) Estimation of UbC-SOD2hp-EGFP gene copy numbers. A 118 bp segment in human ubiquitin C promoter was amplified using a pair of specific primers. Also amplified was human and mouse SOD1 gene using a pair of primers that are complementary to both genes. Both Ubiquitin C and SOD1 are single copy genes. The Ct value of Ubiquitin C was normalized against the SOD1 detected in the same sample. The normalized value from HEK293 genomic DNA represents two copies of the ubiquitin gene. By normalizing this value from mouse genomic DNA samples against the value from HEK293 cells, the estimates of copy numbers of ubiquitin transgene were obtained. The PCR primers used for human and mouse SOD1 gene were 5'-GACCTGGGCAATGTGACTGCTG-3' (forward) and 5'-CACCAGTGTACGGCCAATGATG-3' (reverse); and for ubiquitin C promoter were 5-CGCTGCTCATAAGACTCGGC-3 (forward) and 5-TTTCCTCGCCTGTTCCGCTC-3 (reverse). Bars are average from 4 to 6 animals.
