# Supplementary material for: Pol II–Expressed shRNA Knocks Down Sod2 Gene Expression and Causes Phenotypes of the Gene Knockout in Mice
Source: PLoS Genet. 2006 Jan 27;2(1):e10. doi: 10.1371/journal.pgen.0020010 (PMC1358942; doi:10.1371/journal.pgen.0020010)
Supplement: Figure S3 — (A) Drosha was knocked down by RNAi in the fibroblasts from wild-type and line-8 transgenic mice. The cells were transduced with adenoviral vectors expressing an shRNA against mouse Drosha (shRNA stem sequence: 5′-GGATGAAGATTTAGAGAGTTC-3′). Four days after transduction, the total RNA was extracted from the cells and used for RT-PCR to detect Drosha. The ribosomal RNA L17 was magnified in parallel as input control. The PCR was run for 28 cycles using the following primers for Drosha: 5′-GAGCCTAGAGGAAGCCAAACA-3′ (forward) and 5′-GCCGGACGTGAGTGAAGAT-3′ (reverse); for L17: 5′-CGGTATAATGGTGGAGTTG-3′ (forward) and 5′-ACCCTTAAGTTCAGCGTTACT-3′ (reverse). (B) No EGFP fluorescence could be detected in fibroblasts isolated from line-8 transgenic mice. (C) The same field as in (B) was stained with DAPI. (D) Three days after the fibroblasts were transduced with an adenoviral vector that expressed an shRNA against mouse Drosha, EGFP fluorescence was detected. (E) The same field as in (D) was stained with DAPI. (48 KB PDF) [file pgen.0020010.sg003.pdf]

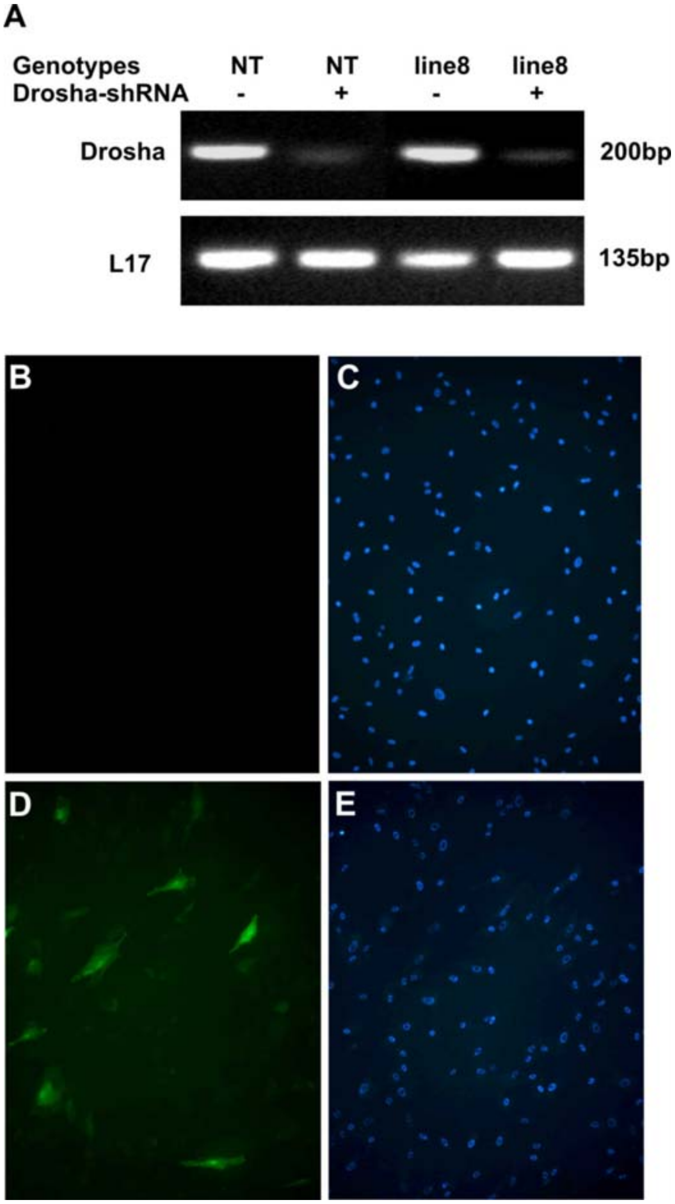

Supplemental figure 3. Silencing Drosha unblocked the expression of EGFP transgene in muscle fibroblasts. (A) Drosha was knocked down by RNAi in the fibroblasts from wild type and line 8 transgenic mice. The cells were transduced with adenoviral vectors expressing an shRNA against mouse Drosha (shRNA stem sequence: 5'-GGATGAAGATTTAGAGAGTTC-3'). Four days after transduction, the total RNA was extracted from the cells and used for RT-PCR to detect Drosha. The ribosomal RNA L17 was magnified in parallel as input control. The PCR was run for 28 cycles using the following primers for Drosha: 5'-GAGCCTAGAGGAAGCCAAACA-3' (forward) and 5'-GCCGGACGTGAGTGAAGAT-3' (reverse); for L17: 5'-CGGTATAATGGTGGAGTTG-3' (forward) and 5'-ACCCTTAAGTTCAGCGTTACT-3' (reverse). (B) No GFP fluorescence could be detected in fibroblasts isolated from line 8 transgenic mice. (C) The same field as in (B) was stained with DAPI. (D) Three days after the fibroblasts were transduced with an adenoviral vector that expressed an shRNA against mouse Drosha, EGFP fluorescence was detected. (E) The same field as in (D) was stained with DAPI.
